# Clinical spectrum and management outcomes of acute febrile illness Among children attending health facilities in northwestern Tanzania, 2020–2021

**DOI:** 10.3389/fped.2026.1799980

**Published:** 2026-03-18

**Authors:** Neema M. Kayange, Oliver Ombeva Malande, Stephan Gehring, Silvia Scialabba, Britta Groendahl, Philip Koliopoulos, Stephen E. Mshana

**Affiliations:** 1 Department of Pediatrics, Weill Bugando School of Medicine, Catholic University of Health and Allied Sciences, Mwanza, Tanzania; 2 Department of Pediatric and Child Health, Bugando Medical Centre, Mwanza, Tanzania; 3 East Africa Centre for Vaccines and Immunization (ECAVI), Kampala, Uganda; 4 Department of Pediatrics & Child Health, Makerere University, Kampala, Uganda; 5 Department of Pediatrics & Child Health, Moi University, Eldoret, Kenya; 6 Department of Pediatrics, University Medical Center of the Johannes Gutenberg University Mainz, Mainz, Germany; 7 Department of Microbiology and Immunology, Weill Bugando School of Medicine, Catholic University of Health and Allied Sciences, Mwanza, Tanzania

**Keywords:** antibiotics, antimalarial, children, fever, management, outcomes, prescribing, Tanzania

## Abstract

**Background:**

The diagnostic challenges of febrile illness in children in low-resource settings and the risks of empirical overtreatment. We evaluated the range of clinical presentations and management outcomes in a cohort of children with acute febrile illness, building on our previous examination of the etiology of these illnesses.

**Methods:**

This prospective cohort study enrolled children aged 1 to ≤12 years who were cared for by attending clinicians across primary, secondary, and tertiary healthcare settings. Management decisions were based on clinical presentation and laboratory and radiographic findings available on the day of enrollment. Outcomes were measured on days 7, 14, and 28. The study also analyzed prescription patterns for antibiotics and antimalarials in relation to established guidelines.

**Results:**

In this cohort of 434 children with acute febrile illness, the most common initial diagnoses were acute respiratory infections (31.3%, 136/434), of which upper respiratory tract infection (URTI) was observed in 57.0% (77/136) and pneumonia in 43.0% (59/136), followed by malaria (23.7%, 103/434). Antibacterial agents were prescribed to 65.3% (284/434) of children. Antibiotic overprescription was observed in 29.6% (84/285) of study participants. Antimalarial drugs were prescribed to 38.9% (169/434) of patients, including 103 patients judged to have malaria by a positive MRDT or a positive blood smear. A total of 66 (39.0%) patients who received antimalarial drugs were negative for either MRDT or blood smear. Fever resolved in 398 children (96.0%, 386/402) by day 28 of follow-up. The most commonly documented complications among admitted children included anemia (36.0%), dehydration (9.1%), shock (8.5%), and acute kidney injury (8.5%). Overall mortality at day 28 was 1.0% (4/434).

**Conclusion:**

In environments with limited diagnostic resources, children with acute febrile illness are often treated empirically. This results in significant overprescription of antibiotics and antimalarials. While short-term results are usually positive, such practices raise concerns about antimicrobial resistance and adherence to guidelines. Better access to point-of-care diagnostics can help decrease inappropriate prescriptions and improve care quality.

## 1 Introduction

Improvements in malaria diagnosis and management, such as the introduction of malaria rapid diagnostic tests and artemisinin combination therapy, have had a significant impact in resource-limited settings, leading to a reduction in irrational use of antimalarial drugs (1, 2). However, diagnostic tools for other infectious conditions that present with similar symptoms, such as viral respiratory infections and mosquito-borne viral infections, continue to pose a significant challenge to implementing effective guidelines and managing cases of acute febrile illness (3, 4). The World Health Organization and the Integrated Management of Childhood Illness (IMCI) program recommend that children with non-severe fever and negative malaria tests should be observed and monitored without the use of antibiotics (2, 5). In many low- and middle-income countries, following guidelines is challenging due to the lack of reliable diagnostic tools to determine the cause of fever in children. Healthcare providers must navigate various pressures, such as caregiver expectations, limited clinical infrastructure, and the concern of missing serious bacterial infections (5–7). Consequently, antimalarials and antibiotics are often prescribed without necessity, leading to the development of antimicrobial resistance (8–10).

A recent large multicenter study conducted across 7 sites in Sub-Saharan Africa and South Asia identified common causes of death in children below five years of age with infection reported be part of the causal chain in 549 of 632 deaths (86.9%) (11). These acute infections can be treated effectively when supported by targeted case management, accurate diagnosis, and proper follow-up. Effective case management is essential for reducing mortality and morbidity and for improving the survival of this vulnerable population (12–14). Management and treatment of AFI depend on various factors, including the patient's clinical condition and the underlying cause of the disease, which can range from bacterial and viral infections to zoonotic diseases such as mosquito-borne viral infections, as well as the challenges posed by limited treatment options (9, 15). The complexity of various cause categories leads to different treatment approaches. These approaches include reviewing clinical history, conducting physical examinations, and performing tests such as blood tests, urine and stool analyses, and specialized laboratory tests, including serology and molecular diagnostics for specific pathogens (16–18. Studies of AFI etiology found that acute respiratory viral infections were the major cause of fever and therefore treatment with antimalarials and antibiotics would not have been necessary (16). The World Health Organization recommended the use of supportive therapy while awaiting results in patients with uncomplicated acute febrile illness (2). These therapies include maintaining adequate rehydration, managing fever and pain, and ensuring rest, which are more specific for viral infections. Patients with AFI need to be monitored closely for any worsening symptoms such as difficulty breathing and persistent vomiting (17, 18). As this may be a sign of a serious illness and can lead to further complications if left untreated, prompt intervention may be needed (17, 19). Guidelines for managing common Severe AFI, such as localizing infections like acute gastroenteritis, pneumonia, urinary tract infections, and malaria, are well outlined in a step-by-step approach. However, management of other causes of acute febrile illness remains less clear, leading to diagnostic and clinical management uncertainties. As a result, clinicians in low and middle-income countries often prescribe empirical antibiotics that may be unnecessary or ineffective. A study in Mwanza reported that approximately 76% of pediatric patients were overprescribed antimalarials. This study aimed to evaluate the clinical spectrum and management outcomes of acute febrile illnesses in children presenting to five health facilities in Mwanza, Tanzania.

## 2 Methods

### 2.1 Study design and population

A prospective cohort study was conducted across five health facilities in Northwestern Tanzania, as previously described (20), children were eligible if they had a fever (≥38 °C) and at least one of the following symptoms: 1) fever lasting less than 7 days; 2) vomiting; 3) headache; 4) rash; or 5) joint pain. Patients with incomplete data or in critical condition requiring intensive care (such as trauma or acute injury) were excluded from the study.

### 2.2 Study setting/region

Part of this cohort has been described in a our previously publication titled “non-malarial etiology of acute febrile episodes in children attending five healthcare facilities in Mwanza, Tanzania years 2020–2021” (20). The fi*ve* sites in which data were collected includes Bugando Medical Centre (BMC), Sekou Toure Region Referral Hospital (STRRH); BHC, Buzuruga Health Centre (BHC), Nyamagana District Hospital NDH and SDDH, Sengerema Designated District Hospital (SDDH). According to the 2022 national census, the region had a population of 3,699,872, compared to 2,772,509 in the 2012 census. Mwanza Region is the second most populous region in Tanzania, and all five health facilities are located within it (Tanzania Health Data Survey 2022) (21).

### 2.3 Follow-up and outcomes

All children with acute febrile illness were followed to determine the management outcome. Clinical disease spectrum, diagnostic practices, prescribing practices in relation to antibiotics and antimalarials, and clinical outcomes at weeks 1, 2, and 4 were assessed. All participants were followed by phone, in addition to hospital chart reviews. Informed written consent was obtained from caregivers, and assent was additionally sought from children aged 7 years and above.

### 2.4 Data collection

During the data collection period, research team from all five health facilities was trained on protocol standard operating procedures focused on data collection procedures and patient documentation of the baseline clinical management on the day of enrollment. Data were collected using a structured questionnaire during enrollment, follow-up, and retrospective hospital chart reviews.

Data collected on the day of enrollment included sociodemographic details (age, sex), signs and symptoms, treatments before and during presentation, illness duration, physical examination findings, immunization history, laboratory results, type of imaging and their results and clinical diagnoses.

During follow-up, data collected included the child's status, fever resolution or persistent readmissions, and any reported complications inpatients. The study team, in collaboration with caregivers, gathered clinical outcome data through mobile phone interviews. Retrospective medical records were collected at the facility level, covering patients' characteristics, admission status, medications given, duration of hospital stays, clinical observations, and referrals to higher-level facilities. This data was documented in paper forms at district and health centers (extracted from patient books) and electronic health records at tertiary and regional hospitals, in accordance with the study protocol.

### 2.5 Clinical and laboratory investigations

The research team conducted a history and physical examination. Laboratory investigations were performed according to specific hospital standard operating procedures. Routine investigations that were performed on children presenting with acute fever at a tertiary-level hospital (BMC) and a secondary-level hospital (STRRH) included: malaria rapid test, malaria microscopy, complete blood count (CBC), human immunodeficiency virus (HIV) testing, stool microscopy, and urinalysis (both dipstick and microscopy). Blood and urine cultures were performed subject to the availability of the reagents and consumables and at the clinician's discretion, based on the case presentation. The decision to perform Chest radiography (CXR) followed the indications prescribed in the national Standard Treatment guidelines subject to the availability of radiological services. For primary-level hospital districts and health centers, routine investigations included malaria rapid tests, malaria microscopy, human immunodeficiency virus (HIV), stool microscopy, and urinalysis (both dipstick and microscopy) when available.

Diagnostic investigations conducted for each participant, according to the study protocol, included a rapid malaria test, a rapid dengue test, malaria microscopy, CBC, urinalysis, blood and urine cultures, and multiplex PCR targeting arboviral infections (blood). Additionally, singleplex PCR for dengue was performed. For participants with flu-like symptoms, multiplex PCR to detect respiratory pathogens was also performed using throat swabs sample.

### 2.6 Diagnosis and management

The diagnosis and clinical management of the study participants were outlined in accordance with the case management protocol detailed in Table 1.

**Table 1:** Study definitions.

Condition	Definition
Fever	Core body temperature of ≥38 °C
Malaria	A positive result from any of the following: malaria rapid diagnostic test (RDT), slide microscopy, or genus-specific malaria PCR at baseline and follow up
Presumptive acute arboviral infection	Positive RDT [e.g., Dengue Non-structural protein 1 (NS1) antigen]
Confirmed arboviral infection	Positive PCR results for arboviruses such as Dengue, Chikungunya, Yellow Fever, Zika, West Nile, Rift Valley, or O’nyong-nyong, or positive anti-arbovirus Immunoglobulin M (IgM) ELISA.
Bloodstream infections (bacteremia/candidemia)	Positive blood culture with pathogenic bacteria or yeast
Urinary tract infections (UTI)	Urine culture showing significant bacteriuria (≥10^5^ colony-forming units/mL).
Acute respiratory infection	Presentation with at least one respiratory sign or symptom lasting ±7 days and confined to the respiratory tract (upper or lower).
Pneumonia	History of cough and/or difficulty breathing, along with one or more of these signs: tachypnea, chest abnormalities, abnormal auscultation, or radiographic findings suggestive of pneumonia.
Sepsis	Systemic inflammatory response syndrome with suspected or confirmed infection, or signs of organ dysfunction.
Anemia	Hematocrit below age-specific cut-off (age: 2 months: <28%; 3–6 months: <29%; 7–24 months: <33%; 25 months-6 years: <34%; 7–12 years: <35%)
Meningitis	Clinical signs (e.g., stiff neck, meningeal signs) and/or cerebrospinal fluid abnormalities consistent with meningitis confirmed by clinicians
Acute gastroenteritis	Presentation with diarrhea, defined as ≥3 loose or liquid stools per day, lasting less than 7 days, reported at any time within the 7 days before enrollment.
Undifferentiated fever	No source of fever was identified following clinical assessment and all available laboratory tests.
**Outcomes**	**Definition**
Resolved Fever	No fever observed for 2 consecutive days before day 7 (± 1), day 14 (± 1), and day 28 (± 1), as reported by caregivers or with a measured temperature ranging from 36.4 °C to 37.5 °C.
Persistent Fever	Fever episode within the 2 days prior to day 7 (± 1), day 14 (± 1), 28 (±1) as reported by caregivers or confirmed by a measured temperature of ≥38 °C
Relapse	Fever was reported on day 14 (± 1) in a child who had previously experienced fever resolution around day 7 (± 1), possibly linked to the initial febrile illness.
Hospitalization	Hospital admission for treatment linked to the febrile illness presented.
Death	Mortality within 28 days (± 1 day) of follow-up, considered potentially related to the initial febrile illness, as evaluated by the attending clinicians.

All participants' laboratory results except multiplex PCR for arboviral infections, urine and blood cultures, and PCR for Nasopharyngeal swabs, were provided to the attending clinicians on the day of enrollment to assist patients' management. Children were seen as either outpatient or inpatients. Initial treatments, including types of antibiotics, antimalarials, antipyretics, and pain relief, were documented by the study team. Antibiotic use as indicated by positive urinalysis, a full blood count suggesting sepsis (elevated WBC count above the normal range of 4.0–11.0 × 10^9^/L, with a high neutrophil count >9 × 10^9^/L) or a clinical diagnosis such as acute tonsillitis, otitis media, or pneumonia, following Tanzania national guidelines, WHO standards, and IMCI protocols was considered appropriate (21–23).

All enrolled children were followed up; children who were not admitted were followed via phone calls on days 7 (± 1), 14, and 28, depending on clinical and laboratory findings. Participants with positive culture results were asked to return to the facility for further management. The study considered the clinical conditions listed in Table 1, these conditions were defined according to the Tanzania Ministry of Health's Standard Treatment guidelines (2021) (21), adapted from World Health Organization (WHO) guidelines for the management of common childhood illnesses (2013) (23). Laboratory confirmation was required for study-specific definition of malaria, bloodstream infections, urinary tract infections (UTIs), and anemia. Febrile participants with no identifiable source of infection based on available clinical and laboratory investigations were classified as having undifferentiated fever.

### 2.7 Data analysis

Data were double-entered into Microsoft Excel and analyzed with STATA version 15 (24). Categorical variables, including sociodemographic characteristics and clinical diagnoses, treatments, and outcomes, were summarized using frequencies and percentages. Quantitative data, including child age, sex, duration of illness, and hospital stay, were summarized using the median and interquartile range (IQR). Differences in demographic and clinical characteristics, diagnoses, prescribed treatments (per Tanzania guidelines), and outcomes between lower-tier (health centers) and higher-tier (tertiary hospital) facilities were evaluated using the Pearson Chi-square test or Fisher's exact test, as appropriate. A *p*-value < 0.05 was considered statistically significant.

## 3 Results

### 3.1 Sociodemographic characteristics of study participants

**Figure 1:**
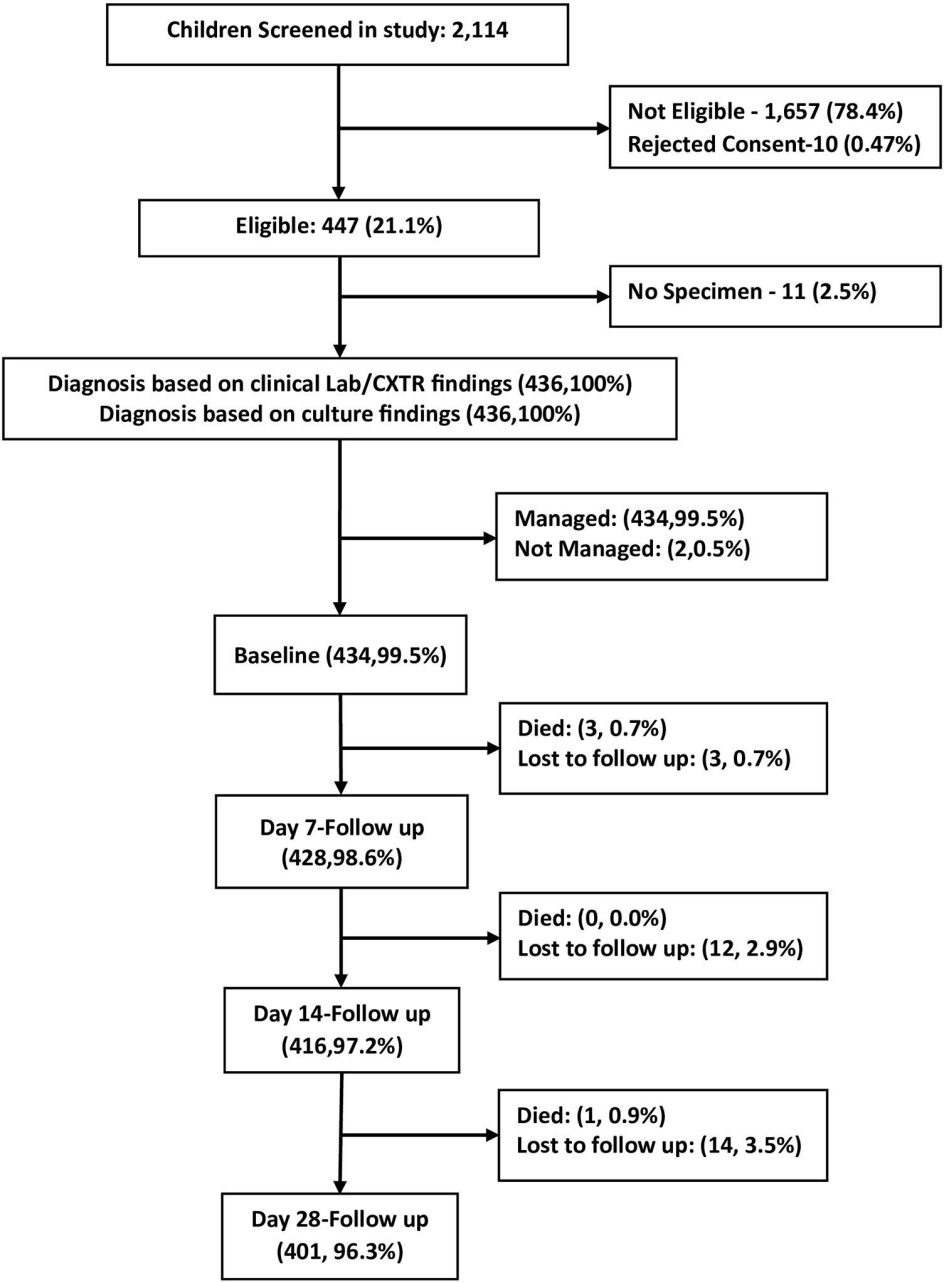
Flow chart of the study participants to health facilities in Mwanza, Tanzania (2020–2021).

During the study period, a total of 2,114 children were screened, and 527 met the eligibility criteria. Of those, 21 were excluded. A total of 436 were included in the final analysis (Figure 1). Of the 436 participants, 56.4% were male. Detailed sociodemographic and clinical characteristics were described previously (20). Of the enrolled children, 335(76.8%) were from four urban hospitals (BMC, SRRH, NDH, BHC) and 101(23.2%) were from one rural hospital (SD).

### 3.2 Spectrum of clinical presentations

The study's definitions indicated that the primary diagnoses among enrolled children, based on outpatient and inpatient charts, were malaria, acute respiratory infections, urinary tract infections, and acute gastroenteritis. According to WHO criteria, 31.2% (136/436) of children were diagnosed with acute respiratory infections, with 56.6% (77/136) having upper respiratory tract infections and 43.4% (59/136) diagnosed with pneumonia. Malaria was identified in 45% (197/436) of cases. Additional clinical diagnoses are detailed in Table 2, and some conditions requiring empirical antibiotics are listed in Table 3.

**Table 2:** Infections clinical diagnosed among children with acute febrile illness at five health facilities in Mwanza, Tanzania (years 2020–2021).

Clinical diagnosed infections (chart record clinic & wards)	Health Facility					Total
	Bugando	Sekou Toure	Buzuruga	Nyamagana	Sengerema	
^a^Malaria	43	38	31	30	27	169
^a^Suspected septicemia	11	7	15	16	9	58
^a^Gastroenteritis	9	11	15	17	21	73
URTI	14	12	10	1	21	77
Unspecified LRTIs	11	1	1	0	5	18
^a^Meningitis	14	0	1	1	1	17
^a^Acute Tonsilitis	4	2	0	4	3	13
^a^Typhoid	4	5	0	2	2	13
^a^Otitis Media	0	0	0	2	0	2
^a^Amoeba	1	1	0	0	0	2
Undifferentiated fever	5	1	3	2	7	18
^a^Urinary Tract Infections	14	18	21	22	23	98
Pneumonia	10	18	10	8	13	59

URTI, Upper Respiratory Tract Infections; LRTI, Lower Respiratory Infections.

a Diagnoses made based on clinical signs and symptoms and hospital records at initial management unless otherwise specified.

**Table 3:** Clinical infections required empirical antibiotics among children with acute febrile illness at five health facilities in Mwanza, Tanzania (years 2020–2021).

Clinical diagnosed infections (chart record clinic & wards)	Health facility					Total
	Bugando	Sekou Toure	Buzuruga	Nyamagana	Sengerema	
^a^Suspected septicemia	11	7	15	16	9	58
^a^Unspecified LRTIs	11	1	1	0	5	18
^a^Meningitis	14	0	1	1	1	17
^a^Acute Tonsilitis	4	2	0	4	3	13
^a^Typhoid	4	5	0	2	2	13
Otitis Media	0	0	0	2	0	2
^a^Amoeba	1	1	0	0	0	2
^b^Undifferentiated fever	5	1	3	2	7	18
^a^Pneumonia	10	18	10	8	13	59
Total						200

LRTI, Lower Respiratory Infections;.

a Diagnoses made based on clinical signs and symptoms at initial management unless otherwise specified.

b Some cases remained with no identified source of fever despite clinical and laboratory investigations were conducted.

### 3.3 Laboratory diagnosis and confirmatory diagnosis

Among the 103 patients with a diagnosis of malaria, 67 (65.0%) tested positive with MRDT, while 36 (35.0%) had a positive malaria blood smear, with a mean parasite count of ± SD (30.8 ± 40.2). Overall, PCR testing detected *Plasmodium falciparum* in 12.0% (52/436) of the study participants (Table 4). Among the 59 patients with pneumonia, 22.0% (13/56) showed indications for chest x-rays, and 61.5% (8/13) of these x-rays were performed. Blood cultures were positive in 6.7% (4/59) of pneumonia cases. No participant tested PCR-positive for any arboviral pathogens, as previously noted (20).

**Table 4:** Infections diagnosed by laboratory investigations among children with acute febrile illness at five health facilities in Mwanza, Tanzania (years 2020–2021).

Clinical Diagnosed Infections (Chart record Clinic & Wards)	Health Facility					Total
	Bugando	Sekou Toure	Buzuruga	Nyamagana	Sengerema	
Laboratory Diagnosed (by Study Tests)						
^d^URTI or colonization	6	4	7	9	7	33
^c^Bacteriuria/Candidemia	7	13	11	9	7	47
^b^Malaria by MRDT	22	20	11	5	9	66
^b^Malaria by BS for mps	7	5	8	11	5	36
^c^Bacteremia	6	4	8	6	2	26
^b^Presumptive Dengue by Rapid test	12	3	3	6	10	34
^d^Arbovirus Infections by PCR	0	0	0	0	0	0
Pearson *χ*^2^ (56) = 114.9218 Pr < 0.001						

URTI, Upper respiratory tract infections; LRTI, lower respiratory infections;.

Children from whom samples were obtained for testing -a. Blood smear microscopy (*N* = 436); b. Blood culture (*N* = 436); c. Urine culture (*N* = 436);.

a Diagnoses made based on clinical investigations at initial management unless otherwise specified.

b Diagnosis made at initial presentation based on laboratory investigations.

c Diagnosis made during the follow-up, based on culture results.

d Diagnosis made retrospectively, using PCR testing results.

### 3.4 Clinical management

The management was done by specialists at BMC and SRRH while in district hospitals (SD and NDH), and Health centre (BHC) patients were managed by medical officers an clinical officers. Of the 436 children enrolled, 356 were treated as inpatients and 78 as outpatients. Two children left before receiving initial treatment. Among these patients, 27.8% received pre-medication prior to hospital arrival, with antipyretics/analgesics (Paracetamol, Ibuprofen and Diclofenac) being the most frequently reported medications. Details on medications, clinical management, and prescription patterns are summarized in Table 5. Antimalarial drugs were prescribed to 38.9% (169/434) of the patients, with 103 diagnosed with malaria based on a positive MRDT or blood smear. Additionally, 66 patients (39.0%) who received antimalarial treatment tested negative on both MRDT and blood smear. The malaria management protocol across all study sites for uncomplicated malaria was outpatient-based, prescribing artemether-lumefantrine twice daily for three days. In case of complicated malaria, artesunate was administered intravenously in three doses within 24 h (at admission, 12-hour and 24 h). With improvement, treatment was then switched to oral artemether-lumefantrine twice daily for three more days, as recommended in the Tanzania and WHO guidelines (21, 22).

**Table 5:** Initial clinical management, antibacterial and antimalarial prescriptions and adherence to guidelines Among children with acute febrile illness presented at health facilities in Mwanza, Tanzania (years 2020–2021).

Characteristics	Health Facility *n* (%) Total 434					Total	*p*-value
Initial Management	Bugando (*N* = 92)	Sekou Toure (*N* = 70)	Buzuruga (*N* = 86)	Nyamagana (*N* = 85)	Sengerema (*N* = 101)		
Managed as inpatients	79 (85.9)	0 (0)	0 (0)	0 (0)	0 (0)	79	
Managed as outpatient	13 (14.1)	67 (95.7)	73 (84.9)	72 (84.7)	97 (96.0)	322	0.001
Referred to higher-level care	0 (0.0)	3 (4.3)	13 (15.1)	13 (15.3)	4 (4.0)	33	
Prescriptions and adherence with guidelines							
Antibiotic Prescriptions							
^a^Prescribed antibiotics	86 (93.5)	48 (68.6)	51 (59.3)	51 (60.0)	48 (47.5)	284	0.001
Prescribed antibiotic with empirical clinical indication	59 (29.5)	26 (44.7)	39 (56.3)	37 (53.5)	39 (43.4)	200	0.001
^b^Prescribed without indication	27 (52.0)	22 (47.0)	12 (47.2)	14 (48.4)	9 (47.2)	84	0.001
Antimalarial Prescriptions							
Prescribed antimalarials	43 (46.7)	38 (54.3)	31 (36.1)	30 (35.3)	27 (26.7)	169	0.102
Prescribed antimalarials with clinical indication	25 (24.3)	23 (22.3)	19 (18.4)	19 (18.4)	17 (16.5)	103	
^b^Prescribed without laboratory indication	18 (28.8)	15 (22.7)	12 (18.2)	11 (16.7)	10 (13.6)	66	0.055

a Prescription reflects initial clinical management decisions made as suggested by Tanzania National Treatment Guideline2017, irrespective of culture results.

b Overprescription refers to medications prescribed at initial management without clinical and/or laboratory indication as per guidelines, and no pathogen identified on culture as well.

1. **Among children, without indication for antibacterial treatment according to national guidelines.

2. **Among children without indication for antimalarial treatment (without laboratory confirmation of malaria).

In total, 76.3% (333/434) of patients had examinations that ruled out malaria as the cause of the current fever, so they were classified as having non-malarial febrile illness. Diagnostic clarification of the clinical picture and the pathogen causing the fever was usually carried out by history and physical examination, with MRDT at the district hospital (NDH, SDDH) and the Health Center (BHC). In some cases, additional laboratory tests, such as urine or stool microscopy, were performed. At the tertiary hospital (BMC), laboratory tests included blood smear, MRDT, urine, stool, culture, and complete blood counts (CBC). At the regional hospital (SRRH), laboratory tests included MRDT, CBC, urine and stool, with occasional blood culture and urine culture.

Antibacterial agents were prescribed to 284 (65.4%%) of 434 patients. Among inpatients, 75 of 78 (96.4%, 95%CI: 92.3–100) received antibiotics compared to 209 of 356 (57.6%, 95%CI: 52.4–62.7) of those treated as outpatients (*P* < 0.001). At enrollment, 46.0% (200/434) of children were prescribed antibiotics empirically. Indications for empirical use of antibiotic in the study sites included positive urinalysis, elevated blood count (White blood cells) suggesting sepsis, or clinical diagnosis with signs and symptoms requiring empirical treatment such as acute tonsillitis, otitis media or pneumonia. Around 29.6% (84/284) were prescribed antibiotics without clear clinical indication; including children with WHO clinical diagnosis of upper respiratory tract infection and acute gastroenteritis. Among patients who received antibiotics only 9.2% (26/284) had a positive blood culture. A total of 136 of the participants were diagnosed with respiratory infections. All participants diagnosed with pneumonia were treated with antibiotics using intravenous ampicillin and gentamicin, and ceftriaxone for the uncomplicated upper respiratory. Children with upper respiratory tract infections were also treated with antibiotics (including Ampiclox syrup, Cotrimoxazole, and Azithromycin).

### 3.5 Distribution of antibiotics prescribed

**Figure 2:**
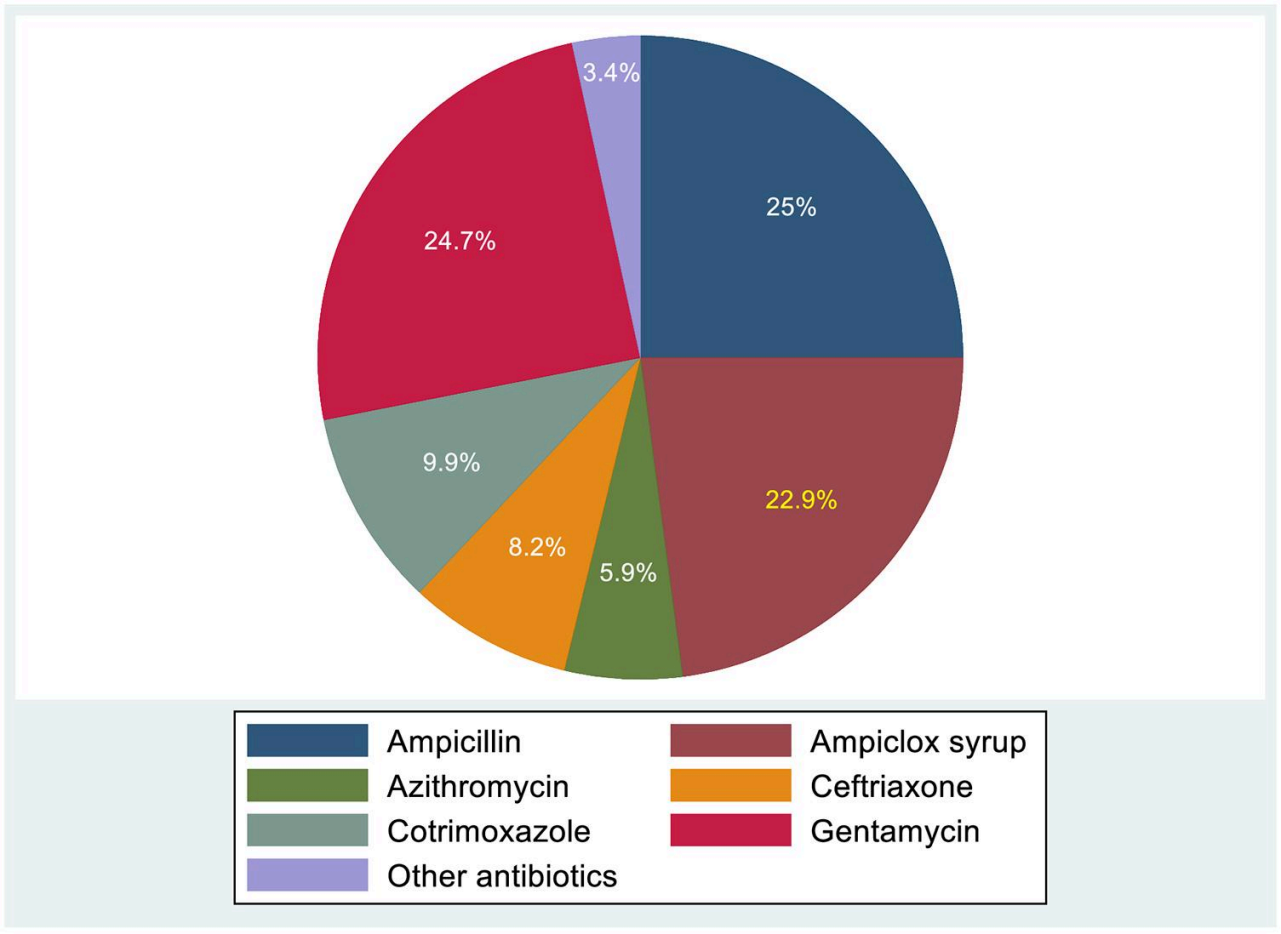
Distribution of antibiotics prescribed among children presenting to health facilities in Mwanza, Tanzania (2020–2021) Key: “Others Antibiotics” include: ciprofloxacin, meropenem, vancomycin, piperacillin-tazobactam and amoxicillin-clavulanic acid.

Among children with acute febrile illness across the five health facilities, 65.7% (284/434) received antibiotics at the initial antibiotic prescription; the medications varied between health facilities. Most children received only one antibiotic agent 65.5% (186/284), while 32.7% (93/284) received a combination of two antibiotics, and 2.1% (6/284) received a combination of three antibiotics. The most commonly prescribed antibiotics were ampicillin (25.0%), gentamicin (24.7%), and ceftriaxone (8.2%) (Figure 2). Additional antibiotics administered to inpatients after culture and sensitivity results included ciprofloxacin, meropenem, vancomycin, and piperacillin-tazobactam.

### 3.6 Clinical outcomes and follow-up

**Figure 3:**
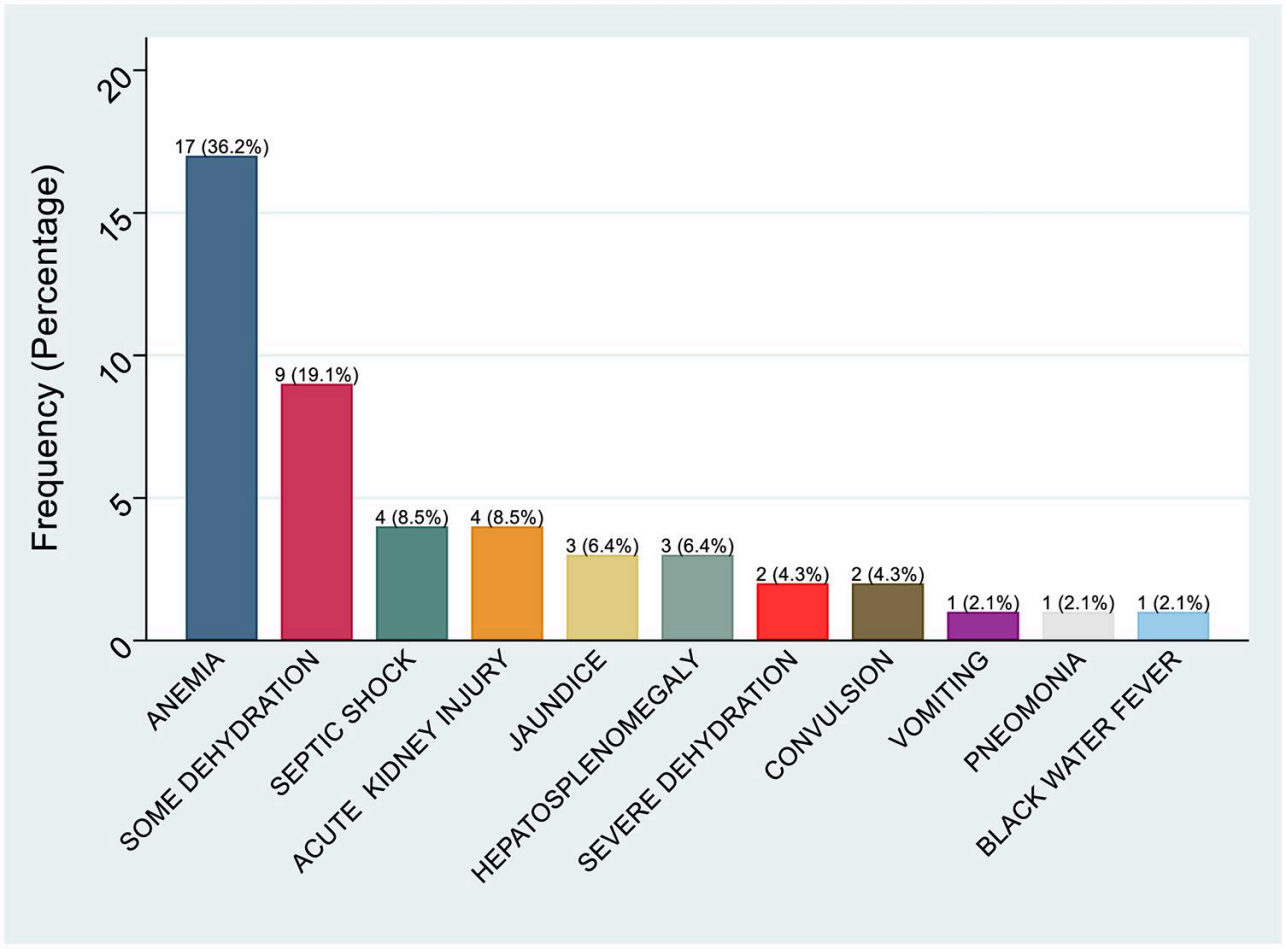
Distributions of complications retrieved from patients records among children attended in health facilities in Mwanza, Tanzania (2020–2021).

All patients were followed by scheduled phone calls on day 7, 14 and 28 after discharge. However, to capture the patients complications for inpatients, patients were followed up at their respective hospitals till discharge. Data was collected by the research team, and additional clinical information, such as complications and blood transfusion records (Figure 3) were retrieved from the hospital electronic records system for patients admitted to tertiary and regional hospitals. A total of 428 participants (98.2%) completed the 7-day follow-up. A total of 7.6% (33/434) of study participants were referred to a higher level of care. Of the 428 participants whose status was known by day 7, 0.7% (3/428) died in hospital. Two died from pneumonia, and one died secondary to acute gastroenteritis complicated with shock and anemia. Three outpatient participants could not be reached and were considered lost to follow-up. Fever had resolved in 398 participants (93.0%). Fever persisted beyond day 7 in only 30 patients. Of the patients contacted on day 14, 416 (95.4%) completed the follow-up, while 12 were not reached; all were outpatients. Fever had resolved in all patients contacted, but 2 patients were readmitted (Table 6).

**Table 6:** Follow-up data in participant presenting with AFI Mwanza 2020–2021.

Follow-up variable	Day 7, *n* (%)	Day 14, *n* (%)	Day 28, *n* (%)
Follow-up Status			
Completed	^a^431	^a^428	^a^402
Lost to follow up	3 (0.3%)	12 (3.0)	14 (3.5)
Child status			
Alive	*N* = 428	*N* = 416	*N* = 402
Died	3 (0.7)	0	1 (0.3%)
Fever Status			
Resolved	398 (93%)	400 (96%)	386 (96%)
Persistent	30	9	0
Relapsed	0	8	0
Hospitalization			
Hospitalized at first visit	78 (18.0%)	0	0
Hospitalized on follow-up	4	16	13
Not hospitalized	356	0	0
Readmission	0	2	0

a Percentages are based on the number of participants reached at each follow-up time point.

Of the 401 participants who completed the day 28 follow-up, one patient died. The cause of death could not be clearly determined; however, according to the parents, the child had persistent fever and continued treatment with traditional medicine. A retrospective review of laboratory results showed that the child tested positive for bocavirus via PCR from a nasopharyngeal swab, but this could not be definitively linked as the cause of death. Overall, there were 4 deaths out of 401 participants (1%) by day 28. Among children with presumed dengue infection based on IgG/IgM serology, fever had resolved in all cases. Out of 434 patients followed, 10.8% (47/434) developed complications, as shown in the Figure 3.

### 3.7 Predictors of hospitalization

Adjusted analysis indicated that children presenting with headaches (OR: 12.97, 95% CI 2.33–72.23, *P* < 0.003), hepatosplenomegaly (OR: 29.17, 95% CI 1.35–630.91, *P* = 0.032), and those clinically diagnosed with meningitis have higher odds of admission (OR: 41.33, 95% CI 2.38–718.12, *P* < 0.011). In the initial analysis, sociodemographic factors such as age 1–5 years, female gender, and symptoms such as fever lasting less than 3 days, along with muscle and joint pain, were associated with admission, but these associations became non-significant after adjusting for other variables (see Supplementary Table S2).

## 4 Discussion

This prospective study analyzed the clinical differential diagnosis patterns and management outcomes of children presenting with acute febrile illness at five healthcare facilities in Tanzania (four urban and one rural). The study found that overprescription of antibiotics and antimalarials occurred in about 29.6% and 39% of cases, respectively. The most common diagnoses were acute respiratory infections, including upper respiratory infections and pneumonia, followed by malaria. Malaria was evenly distributed across all sites but confirmed *Plasmodium falciparum* cases detected through microscopy and PCR were more frequent at Nyamagana District Hospital and Buzuruga Health Centre. The rate of positive *Plasmodium falciparum* tests was lower at tertiary and secondary hospitals, likely because patients first seek care at primary facilities where they may have already received treatments, including antimalarials. None of the children were diagnosed with arboviral infections, possibly due to limited awareness among health workers about dengue and chikungunya in Tanzania, despite reports of outbreaks and studies indicating their presence (25, 26).

Common complications observed in admitted children included anemia (36.0%), dehydration (9.1%), shock (8.5%), and acute kidney injury (8.5%). These were especially frequent in patients diagnosed with malaria and septicemia. It was noted that children with uncomplicated malaria can quickly develop into severe or complicated malaria due to immature immunity. The pathophysiology of severe malaria involves erythrocyte destruction, reduced production, anemia, and sequestration of parasitized red blood cells (pRBCs) in blood vessels (27). Observed complications of malaria in this study, highlighting the importance of emphasizing ongoing primary preventive strategies, such as use of insecticide-treated bed nets and malaria vaccination for all children in moderate and high endemic areas as per WHO recommendation. In addition, secondary measures to prevent severe illness, that include appropriate malaria diagnosisis and effective treatment of uncomplicated cases should be sustained. Additionally, there is a need to improve healthcare workers' understanding regarding monitoring and follow-up of children with high risk of worsening and ensuring strict compliance with protocols guidelines (27–29).

Approximately 39% of children were prescribed antimalarial drugs without diagnostic confirmation by a rapid diagnostic test or microscopy. This is lower than in a previous study conducted in the same area, where a single center reported extensive antimalarial overprescription of about 76.6% among children (4). The findings reported herein are higher, however, than those reported by Kazaura et al. in a study conducted in the Lake Victoria region that found an overprescription rate of 7.0% in 2016, 10 years ago (30). The frequency of prescribing antimalarials without a diagnostic indication may stem from healthcare workers' uncertainty in trusting negative microscopy or MRDT results, as well as the patient's clinical condition (30, 31). Other studies in Ethiopia and Mozambique have reported antimalarial overprescription rates ranging from 7.0% to 72%, respectively (31, 32). The 2021 Tanzania National Guidelines and WHO Guidelines highlighted that malaria testing should be performed with an MRDT before treatment in all health facilities without access to the quality gold-standard malaria slide microscopy (21, 22). This challenge is worsened by the scarcity of diagnostic tools for children with undifferentiated fever, which hampers clinicians' ability to identify causes other than malaria. Moreover, in resource-limited settings, clinicians treating febrile children showing malaria-like symptoms might be more prone to empirically prescribe antimalarials (5, 33). Overprescription of antimalarials raises concerns about the long-term efficacy of malaria treatment and the emergence of drug resistance. Artemisinin resistance markers have been reported in various areas in Tanzania (34), including a recent publication that reported the detection of the Pfk13 R561H mutation, associated with partial artemisinin resistance, in 2 patients enrolled in 2022 in Mwanza, Tanzania (35). This finding is particularly of concerning as it may compromise first-line treatment.

The main indication for prescribing antimicrobials at the initial visit in tertiary and regional hospitals was an elevated white blood cell count, urinalysis findings, and illness severity while awaiting culture results. At the primary health care level, the indication to start antibiotics depended on the clinical presentation of patients and clinicians' judgment, as most investigations were not available, especially in outpatient clinics. Cultures and complete blood count was were not part of routine investigations, therefore, overprescription of antibiotics of 29.6% in this cohort should be interpreted with caution, particularly in light of the above challenges, especially in district hospitals and health centers. Antibiotic prescriptions without indication were most often noted in children with a clinical diagnosis of upper respiratory tract infection and acute gastroenteritis (Supplementary Table S1). As observed in previously, this trend of overprescription was more evident among inpatients at tertiary hospitals (32, 36). Notably, most febrile children who did not receive antimalarials or antibiotics still recovered, suggesting that their illnesses were likely due to other causes, more likely viral infections, which do not respond to antimicrobial or antimalarial therapy (37, 38). This underscores the need for adherence to clinical guidelines, improved diagnostic capacity, better healthcare education, and trust in laboratory results in order to ensure adequate and high-quality care for this vulnerable population.

Fever management remains a major challenge for most clinicians, especially in settings where diagnostic tools are limited. In this study, fever resolved in 92.3% of participants on day 7 of follow-up and in 96.0% by day 28. Children with negative malaria often experienced spontaneous fever resolution supporting obversion from other African studies (34, 37, 38). These findings indicated that there is room to wait before starting antimalarials and antibiotics for children with uncomplicated febrile illness. The mortality rate observed in the current study was 1%. This is consistent with the findings of another study conducted in Ethiopia, which reported a mortality rate of 1.5%. Prompt case detection and appropriate management coupled with follow-up of patients are essential to achieving the Sustainable Development Goal of reducing under-five mortality to less than 25 deaths per 1,000 live births by 2030, particularly for preventable diseases (39, 40).

### 4.1 Strength and limitation

A key strength of the present study is the integration of clinical and laboratory data reported in a previous study using WHO diagnostic algorithms, along with robust follow-up on outcomes. This provides a solid foundation for future qualitative research into the root causes of overprescription of antimalarials and antibiotics and for quality improvement initiatives focused on the more rational use of antibiotics and antimalarials. Nevertheless, the current analysis has potential limitations; i) during follow-up, recall bias and chart review may have affected the reliability of the data, especially for caregiver-reported outcomes, ii) laboratory and diagnostic testing did not cover all known potential infections limiting the information needed to improve overall fever management guidelines, iii) patients' prior treatment history, local antimicrobial resistance patterns, and medication costs could have influenced clinicians' treatment decisions and iv) participants were enrolled in five health facilities; sampling was not random across the region, and other clinical settings were not included. Therefore, selection bias related to facility choice was possible, limiting the generalizability of the findings.

## 5 Conclusion and recommendation

The high rate of children with acute respiratory tract infections (ART) and pneumonia underscores the need for intensified ART management, particularly by promoting early care-seeking behaviour and treatment to reduce hospitalizations. The varying malaria transmission levels across study sites underscore the need to strengthen interventions in high-risk areas, and efficiently allocate resources toward malaria elimination. Notably, about 29.6% of antibiotic prescriptions lacked clinical justification, and 39.0% of children with febrile illnesses received antimalarial drugs despite negative rapid diagnostic tests (MRDT) or blood microscopy results. This underscores the need to improve antimicrobial use by incorporating diagnostic tools to identify common pathogens coupling with antimicrobial stewardship programmes and improved healthcare workers' adherence to guidelines across health facilities at all levels.

## Data Availability

The raw data supporting the conclusions of this article will be made available by the authors, without undue reservation.
