# Supplementary material for: Clinical spectrum and management outcomes of acute febrile illness Among children attending health facilities in northwestern Tanzania, 2020–2021
Source: Front Pediatr. 2026 Mar 18;14:1799980. doi: 10.3389/fped.2026.1799980 (PMC13038892; doi:10.3389/fped.2026.1799980)
Supplement: Supplementary file 1 [file Table1.pdf]

Supplementary Table 1: Infections Clinical Diagnosed against standard treatment among Children with Acute Febrile Illness at Five Health Facilities in Mwanza, Tanzania (2020–2021).

Clinical Diagnosed Infections (Chart record Clinic & Wards)	Total patient	By Diagnostic test cases	Antibiotic and given & antimalaria	Standard guidelines	Overprescription
Clinical malaria	169	103	Ampicillin, Gentamycin, ceftriaxone, cotrimoxazole	Clinical malaria	66/169= 39%
Suspected septicemia	58	26	Ampicillin, Gentamycin, ceftriaxone, cotrimoxazole<br>Azithromycin, meropenem, vancomycin, Ampicillin, Gentamycin, ceftriaxone, cotrimoxazole<br>Azithromycin	Start Empirical antibiotics Treatment	
Gastroenteritis	73	WHO Clinical Diagnosis	Ampicillin, Gentamycin, ceftriaxone, cotrimoxazole<br>Azithromycin	Withhold antibiotics	49/73 x 100 =67%
URTI	77	33	Ampicillin, Gentamycin, ceftriaxone, cotrimoxazole<br>Azithromycin	Withhold antibiotics	38/77 x 100= 49.4%
Unspecified LRTIs (Bronchiolitis)	18	Clinical Diagnosis	Ampicillin, Gentamycin, ceftriaxone, cotrimoxazole	Withhold antibiotics unless suspected co-infection pneumonia	
Meningitis	17	8	Ampicillin, Gentamycin, ceftriaxone, cotrimoxazole	Start Empirical Treatment-3rd Generation Cephalosporin	
Acute Tonsilitis	13	Clinical Diagnosis	Ampicillin, Gentamycin,	Amoxicillin	
Typhoid	13	Clinical Diagnosis plus hospital records	Ampicillin, Gentamycin, ceftriaxone, cotrimoxazole	Ciprofloxacin	

Otitis Media	2	Clinical Diagnosis plus hospital records	Ampicillin, Gentamycin, ceftriaxone, cotrimoxazole	Amoxicillin	
Amoeba	2	Clinical Diagnosis plus hospital records	Ampicillin, Gentamycin, ceftriaxone, cotrimoxazole	Metronidazole	
Undifferentiated fever	18	Clinical Diagnosis	Ampicillin, Gentamycin, ceftriaxone, cotrimoxazole	Start Empirical Treatment on patient clinical status while continue investigation	
Pneumonia	59	WHO clinical diagnosis	Ampicillin, Gentamycin, ceftriaxone, cotrimoxazole	Amoxicillin	
Urinary Tract Infections	89	47-Urinalysis and Urine culture	Ampicillin, Gentamycin, ceftriaxone, cotrimoxazole	Start antibiotics those with positive urinalysis	42/89 x 100 =47.2% Prescription not supported by laboratory test
