# Supplementary material for: Clinical spectrum and management outcomes of acute febrile illness Among children attending health facilities in northwestern Tanzania, 2020–2021
Source: Front Pediatr. 2026 Mar 18;14:1799980. doi: 10.3389/fped.2026.1799980 (PMC13038892; doi:10.3389/fped.2026.1799980)
Supplement: Supplementary file 2 [file Table2.pdf]

Supplementary Table 2; Predictors of Hospitalization among children attending Health facilities in Mwanza 2020-2021.

Variable	Crude			Adjusted		
	**OR**	**95%CI**	**P-value**	**COR**	**95%CI**	**P-value**
**Study site**						
BHC	0.19	(0.09,0.40)	0.001	0.27	(0.06,1.22)	0.089
NDDH	0.14	(0.06,0.31)	0.001	0.09	(0.02,0.56)	0.009
STTRH	0.20	(0.09,0.44)	0.001	0.11	(0.02,0.82)	0.031
SDDH	0.06	(0.02,0.17)	0.001	0.03	(0.00,0.28)	0.002
BMC	1.00			1.00		
**Sex**						
Female	0.59	(0.35,0.98)	0.041	0.65	(0.21,2.06)	0.467
Male	1.00			1.00		
**Age (years)**						
1-5	0.20	(0.08,0.52)	0.001	0.27	(0.01,7.36)	0.441
6-12	1.00					
Fever duration						
1-3 days	0.87	(0.48,1.58)	0.649	0.64	(0.42,1.20)	0.534
4-7 days	1.00					
**Nutritional Status**						
Severely	1.00			0.20	(0.00,9.62)	0.418
Moderately	1.30	(0.41,4.11)	0.654	1.69	(0.21,13.63)	0.624
Mild	0.42	(0.15,1.22)	0.112	0.10	(0.02,0.44)	0.003
Normal	1.00			1.00		
**Vaccination Status**						
Yes	0.21	(0.59,0.74)	0.015	0.22	(0.01,3.91)	0.301

No	1.00			1.00		
**Blood Transfusion**						
Positive	18.12	(6.40,51.25)	0.001	22.65	(1.93,265.60)	0.013
Negative	1.00			1.00		
**Fever duration (days)**						
1-3days	0.37	(0.21,0.65)	0.001	0.37	(0.10,1.44)	0.15
4-7 days	1.00			1.00		
**Vomiting**						
positive	1.66	(1.00,2.74)	0.047	0.84	(0.26,2.70)	0.77
negative	1.00			1.00		
**Diarrhea**						
positive	1.02	(0.60,1.76)	0.944	0.99	(0.88,2.43)	0.092
negative	1.00			1.00		
**Dehydration**						
positive	0.56	(0.30,1.04)	0.068	0.43	(0.58,1.75)	0.103
negative	1.00			1.00		
**Headache**						
positive	6.82	(3.26,14.27)	0.001	12.97	(2.33,72.23)	0.003
negative	1.00			1.00		
**Muscle pain**						
positive	4.15	(1.36,12.73)	0.013	21.77	(2.24,211.20)	0.008
negative	1.00			1.00		
**Joint/bone pain**						
Positive	17.70	(3.60,86.99)	0.001	74.78	(2.91,1924.48)	0.009
Negative/Positive, lumbar	1.00			1.00		
**Pallor**						
Mild/Moderate	7.10	(3.75,13.44)	0.001	3.60	(0.72,17.97)	0.072

No/Severe	1.00			1.00		
**Hepato or Splenomegaly**						
Positive	20.17	(4.20,97.01)	0.001	29.17	(1.35,630.91)	0.032
Negative	1.00			1.00		
**Urinary Tract Infection**						
Positive	4.75	(2.25,9.99)	0.001	1.99	(0.20,20.27)	0.562
Negative	1.00			1.00		
**Meningitis**						
Positive	2.23	(4.21,97.29)	0.001	41.33	(2.38,718.12)	0.011
Negative	1.00			1.00		

OR, odds ratio; CI, confidence interval; ,Poisson regression
